# Impact of asthma on endoscopic sinus surgery outcomes for chronic rhinosinusitis with polyposis - A cohort study

**DOI:** 10.1016/j.amsu.2021.102386

**Published:** 2021-05-14

**Authors:** Meryem Lahjaouj, Mohammed Laachoubi, Khadija EL. Bouhmadi, Youssef Oukessou, Sami Rouadi, Reda Abada, Mohammed Roubal, Mohammed Mahtar

**Affiliations:** a ENT Department, Face and Neck Surgery, Hospital August, 20’1953, University Hospital Center IBN ROCHD, Casablanca, Morocco; b Faculty of Medicine and Pharmacy, Hassan II University of Casablanca, B.P 5696, Casablanca, Morocco

**Keywords:** Nasal polyposis, Asthma, FESS, SNOT 22

## Abstract

**Introduction:**

Chronic rhinosinusitis with polyposis (CRSwNP) is a multifactorial naso-sinusal inflammatory disease that affects 2–4% of the adult population. It highly affects the patient quality of life (QoL) in many levels making it a public health issue. The management of CRSwNP is based on a detailed clinical history, a complete endoscopic examination and a precise computed tomographic (CT) analysis. The aim of this study is to evaluate the prevalence and severity of the various CRS clinical manifestations as well as to highlight the potential relationship between symptom scores, asthma and ESS outcomes.

**Patients and methods:**

A retrospective cohort study was performed in the 20 August hospital, between January 2017 and December 2018, on patients diagnosed with CRS according to guidelines recommendations, and were beforehand refractory to initial medical therapy and elected to FESS. The patients were divided into two groups, the first group (G1) of patients with asthma and the second (G2) without asthma in order to expose an eventual significant difference in the improvement of symptoms after surgery. The Sino Nasal Outcome Test-22 (SNOT-22) was used to evaluate QOL.

**Results:**

A total of 100 patients participated in the study with an average age of 44.53 years. The sex ratio was 1.04 (51% men). Asthma was present in 48% of patients while 20% of patients were intolerant to aspirin with a significant difference between the asthmatic and non-asthmatic group (p < 0.05). It appears that asthma was not objectively correlated with a higher Lund Mackay radiological score (p > 0.05). A higher significant improvement was observed between preoperative and postoperative SNOT-22 scores in group with asthma [42.7 ± 16.3 versus 11.8 ± 9.1] and in group without asthma [38.3 ± 15.1 versus 10.5 ± 14.2].

**Conclusion:**

Asthma in CRS is an additional symptom in these patients, mainly reflected in the subset of nasal symptoms in SNOT-22. However, it did not significantly affect the quality of life of the CRSwNP population.

## Highlights

• Chronic rhinosinusitis with polyposis highly affects the patient quality of life in many levels making it a public health issue.
• The aim of this study is to highlight the potential relationship between symptom scores, asthma and ESS outcomes.
• SNOT-22 scores were significantly reduced in the postoperative period in the asthma and non-asthma groups, with no significant difference between the 2 groups.

## 1 Introduction

Chronic rhinosinusitis (CRS) is a bilateral chronic infalmmatory pathology of the nasal mucosa, which may be associated with edematous degeneration of the nasal mucosa, thus forming polyps. It constitutes a major health problem, since its prevalence reaches 4% of the general population, and its high cost and drop in individual production due to increased absenteeism and the deterioration of the quality of life (QoL) [1,2]. Its management is based on a detailed clinical history, a complete endoscopic examination and a precise computed tomographic (CT) analysis. It can be associated with other diseases such as asthma, aspirin intolerance, cystic fibrosis, responsible for polymorphism at several levels: clinical presentation, treatment and evolution after treatment [3].

SNOT22 represents a clinical means that reflects the degree of severity of the disease as well as its impact on the mood and well being.

Endoscopic endonasal surgery ESS is the most preferred treatment for the CRS forms resistant to medical treatment, it is based on the theory that the diseased nasal mucosa can restore its function after improving ventilation and drainage [4].

The aim of this study is to evaluate the prevalence and severity of various clinical manifestations of CRS as well as to expose the potential relationship between symptom scores, asthma and outcomes following ESS and to analyse the positive effects of ESS on clinical symptoms and QoL. We hypothesized that the patients without asthma may have lower baseline symptom scores than those with asthma.

## 2 Patients and methods

A cohort study was fulfilled in the otorhinolaryngology departement of the 20 August hospital between January 2017 and December 2018.

A serie of 100 adult patients undergoing ESS for medically refractory CRS formed the study group, with 30 patients lost to follow-up. CRS was refractory if symptoms persisted after a minimum of six weeks of treatment which includes antibiotics, topical corticosteroids and antihistamines. Asthma and aspirin intolerance were systematically sought. Preoperatively, the patients were clinically examined and evaluated according to the SNOT22 clinical score. This questionnaire was self-administered in the standard fashion with 22 items graded on a 6-point scale ranging from 0 (no problem) to 5 (problem as bad as can be) with a maximum score of 110 [5], and according to the paranasal CT scan results as per Lund- Mackay score (0: no opacity, 1: partial opacity, 2: total opacity for each sinus) [6].

The study population underwent ESS with a standard technique. The extent of surgery was determined by the severity of disease and extent of involvement of sinuses as per the preoperative CT scan and nasal endoscopy.

The patients were followed up postoperatively with a control at 3 months and at 6 months to determine the effect of asthma on outcomes of ESS on CRS, based on endoscopic examination and SNOT22 scoring.

The study population was divided into 2 groups, the first one (G1) of patients with asthma and the second (G2) of patients without asthma in order to show the presence or not of a significant difference in the improvement of symptoms after surgery.

Data management and analysis were performed using IBM SPSS Statistics for Windows, version 25.0.0 (IBM Corporation, Armonk, NY). Categorical data were summarized as frequencies, and cross-tabulations and ×2 tests for significance made comparisons across allocated groups. Continuous variables were summarized as the mean and range, and comparisons between groups were made using the independent samples *t*-test. All significance tests used a two-sided P-value of 0.05.

The study was reported in line with the STROCSS criteria [7]. And register in open access database (UIN: researchregistry6522).

Ethical approval has been exempted by our institution.

## 3 Results

### 3.1 Demographic and clinical results

A total of 100 patients were enrolled in our study. The sex ratio M/F was 1.04 (men: 51%) and the mean age was 44.53±10 years.

Asthma was presented in 48% of patients while, 20% of patients were intolerant to aspirin. Most of the patients (77%) had never been operated on for endoscopic sinus surgery ESS for CRSwNP, and only 20% of cases presented recurrences, and 3% of them were operated on more than 2 times. The average time between symptoms and the first consult was 5 years with extremes ranged from 6 months to 10 years. Bilateral permanent nasal obstruction was the main symptom in all patients, the rhinorrhea was watery in 35%, purulent in 16%, bloody in 1% while it was absent in 48% of the cases and hyposmia was present in 68% of cases.

The average SNOT 22 score was 41.03, with extremes of 5–69 (Table 1).

**Table 1:** Characteristics of patients with and without asthma.

characteristics	All patients (n = 100)	G1 (n = 48)	G2 (n = 52)	p-value
Age (years)	44,5 +- 10	43,6	42.3	0.62
Gender (mal: %)	51%	18%	33%	0.09
Aspirine intolerance (yes:%)	20%	25%	1%	0
Revision ESS, (%)				
0	77%	73%	84.6%	0.06
1-2	20%	20%	9.6%	0.094
≥ 3	3%	6.7%	5.7%	0.051
Symptoms:				
Nasal obstruction	100%	100%	100%	–
Rhinorrhea	52%	24%	28%	–
hyposmia	68%	34%	35%	–
SNOT22 score, mean (écart-type)	41.03 (14.5)	42.7 (16.3)	38 (15)	0.163
Lund-Mackay score, mean (extreme)	16.8 (7.2)	17.4 (+- 5.8)	16 (6.1)	0.430

Most of the patients (84%) did not respond to a well-conducted medical treatment based on short-course oral corticosteroid therapy and topic corticosteroid therapy for at least 3 months, whether or not combined with antibiotic therapy.

### 3.2 Radiological results

The CT scan of paranasal sinus was performed in 75% of patients. The average Lund-Makay score was 16.8 with extremes of 2–24, the score was ≥15 in 34 cases (45.3%) and ≤14 in 41 patients (54.7%). It also showed anatomical variants, since 4% of patients had a procidence of the optic nerve and carotid canal.

### 3.3 Surgical treatment

All patients underwent an endoscopic sinus surgery ESS with polypectomy combined with a middle meatotomy. Anterior and posterior ethmoidectomy were performed in 85% of the cases, a *trans*-ethmoid sphenoidotomy in 60% of the casess, associated with a lower turbinectomy in 2% of cases.

The result after ESS were evaluated with SNOT22 at three and six months (Table 2).

**Table 2:** Evaluation of SNOT22 after surgery at 3 and at 6 months.

	G1	G2	p-value
SNOT 22 preop (mean ± SD)	42.7 ± 16.3	38.3 ± 15.1	0.451
SNOT 22 at 3 months (mean ± SD)	16 ± 10.4	16.6 ± 14.8	0.873
SNOT22 at 6 months (mean ± SD)	11.8 ± 9.1	10.5 ± 14.2	0.753

### 3.4 Complications

In our series we did not have major complications and postoperative synechiae was the most common problem, encountered in 12 (12%) patients. These were easily cleared by systematic postoperative outpatient care with meticulous cleaning of the nasal cavity. Minor complications like nasal bleeding were occasionally encountered in eight (10%) patients who were treated conservatively with packing and did not require blood transfusion.

## 4 Discussion

Chronic rhinosinusitis with polyposis (CRSwNP) is a multifactorial naso-sinusal inflammatory disease that occurs in 2–4% of the adult population [8]. It is often associated with damage of the lower respiratory tract. It is associated with an important impact on quality of life (QoL) and a heavy financial burden, since it represents a major cause of absenteeism and decrease in productivity in the workplace.

In our serie, asthma was present in 48% of the patients which is similar to the literature, where it varies between 20 and 60% [9]. Females present a greater proportion of CRSwNP in combination with asthma [10]. In the present study there was no significant difference in terms of gender between the asthmatic and non-asthmatic groups (p > 0.05).

The association with asthma is not in itself a factor of clinical severity of naso-sinusal polyposis [11,12]. In our study, no significant difference in terms of nasal obstruction, anosmia and rhinorrhea between the asthmatic and non-asthmatic groups (p > 0.05). Previous studies have established a link between the diagnosis of asthma and a greater severity of CRS disease. In particular, asthmatic patients have been shown to have poorer radiographic CRS disease severity based on Lund-MacKay scores [13], and those with worse asthma severity have higher Lund-MacKay scores [14]. In our study asthma was not objectively correlated with a higher Lund Mackay radiological score (p > 0.05). According to the literature, the diagnosis of asthma was not predictive of severe nasosinusal polyposis if asthma is well controlled [12].

Widal Syndrome should be checked in all patients with nasosinusal polyposis. Aspirin intolerance, found in more than half of all patients (CRSwNP) associated with asthma, was a provider of a clinically more severe polyposis [15,16]. Recent studies have suggested that aspirin desensitisation in patients suffering from Widal syndrome would have the clinical effect of improving respiratory as well as rhinological signs [15,17]. In our study aspirin intolerance was present in 20% of the patients with a significant difference between the asthmatic and non-asthmatic group (p < 0.05).

Medical treatment remains the reference treatment. As asthma associated with CRSwNP is highly eosinophilic, anti-leukotriene (Montelukast), anti-IgE (Omalizumab) and anti IL-5 (Mepolizumab and reslizumab) treatments have shown promising results for both asthma and polyposis-sinus [18,19].

FESS was introduced in the 1960s by Professors Messerklinger and Wigand. It was popularised in Europe by Stammberger, then in North America by Kennedy [20]. ESS is designed to maintain physiological function and anatomical structure by restoring sinus drainage and ultimately improving mucociliary function of the sinuses. It should be reserved for patients with corticosteroid resistance and/or corticosteroid dependence and for patients presenting with a contraindication to general corticosteroid therapy. In the literature, radical ethmoidectomy seems to give a better postoperative result from an endoscopic and olfactory point of view than functional ethmoidectomy, but not on other rhinological signs or respiratory function [11]. Naso-sinusal polyposis in asthmatic patients was characterised by a post-operative recurrence rate of 20%. The association with asthma appears to be a predictor of recurrence after nasosinus surgery [21,22]. In the present study there was no significant difference in terms of recurrence after nasosinus surgery between the asthmatic and non-asthmatic group (p > 0.05). A prior sinus surgery appeared to indicate a poor prognosis after FESS. Most studies have shown the same result [22,23]. Revision surgery is considered more difficult because of the lack of landmarks which may increase the risk of the surgical procedure and its complications [24].

A number of disease specific questionnaires have been developed to evaluate quality of life. SNOT-22 is the most widely used and validated questionnaire [25,26]. We also used the same instrument for assessment of quality of life of patients after surgery.

The CRSwNP has already been associated with poorer outcomes and reduced quality of life. Further analysis of the CRSwNP population did not find a significant association between atopy and measures of quality of life. Thus, the presence of atopy in CRSwNP appears to have no significant impact on the symptom burden of CRSwNP [27,28].

In our study, we observed that SNOT-22 scores were higher in the preoperative period, then reduced significantly in the post-operative period in the asthmatic and non-asthmatic group without significant difference at the 3rd month and the 6th month respectively (p > 0.05).

This study has several limitations that should be acknowledged. The sample size was small; 30 patients were lost to follow-up, and the conclusions cannot be taken for granted.

But the strengths of this study include the presence of a control group, the use of a properly adapted and validated assessment instrument, the assessment of results done from the standpoint of the patient, and a follow-up to 6 months. Furthermore, surgeons were blinded for preoperative SNOT-22 score.

This study has served as a guideline for further research in the future and cannot be generalised to the entire current FESS population. More extensive studies will be needed.

## 5 Conclusion

Asthma in CRS causes an additional symptom in these patients, which is mainly reflected in the subset of nasal symptoms in SNOT-22. However, it did not significantly affect the quality of life of the CRSwNP population.

Although there has been major advances in this field, there is still a lack of consistent evidence to reach firm conclusions about the relationship between CRS and asthma. Research on the basic pathophysiology of the nose and demonstration of the unified airway concept are mandatory. Clarification is also required concerning whether CRS management affects other comorbid lower airway diseases. A collaboration between otorinolaryngologist and pneumologist is necessary for a global and adequate management of the asthmatic patient with naso-sinusal polyposis.

## Ethical approval

This research did not receive any specific grant(s) from funding agencies in the public, commercial, on non-for-profit sectors.

## Sources of funding

This research did not receive any specific grant(s) from funding agencies in the public, commercial, on non-for-profit sectors.

## Author contribution

Lahjaouj Meryem: study concept and acquisition of data.

Laachoubi Mohammed: Corresponding author, writing the paper.

El bouhmadi Khadija: writing the paper.

Youssef Oukessou: study concept.

Sami Rouadi: supervision.

Reda Abada: revising the paper.

Mohamed Roubal: revising the paper.

Mohamed Mahtar: final approval.

## Research Registration Unique Identifying Number (UIN)

Researchregistry: 6522.

## Guarantor

Laachoubi Mohammed.

## Consent

Written informed consent was obtained from the patient for publication of this research study. A copy of the written consent of each patient is available for review by the Editor-in-Chief of this journal on request.

## Provenance and peer review

Not commissioned, externally peer-revieweed

## Declaration of competing interest

The authors declare no conflicts of interest for this article.
